# Q&A: Who needs a centrosome?

**DOI:** 10.1186/1741-7007-11-28

**Published:** 2013-04-11

**Authors:** Mónica Bettencourt-Dias

**Affiliations:** 1 Instituto Gulbenkian de Ciência, Rua da Quinta Grande 6,, Oeiras, 2780-156, Portugal

## What is the function of the centrosome?

The centrosome has several functions. The central one is as the major microtubule organizing center (MTOC) in proliferating animal cells: thus, it helps to organize the microtubules that form the mitotic spindle in dividing cells, and orchestrate a wide variety of cellular processes, including cell motility, signaling, adhesion, coordination of protein trafficking by the microtubule cytoskeleton and the acquisition of polarity. The centrosome has crucial links to the nucleus, the Golgi, cell to cell junctions and acto-myosin cytoskeleton that are very important in positioning it and thus shaping the microtubule cytoskeleton in relation to the cell and the organism (reviewed in [1]). The role of the centrosome in organizing cellular microtubules can differ from cell to cell and be regulated differently in different phases of the life of a cell.

## How does the centrosome perform its ‘organizing’ function?

By stimulating the formation of microtubules and anchoring them. Microtubules are dynamic structures formed by polymerization of tubulin. In dividing cells, for example, the microtubule cytoskeleton is continuously reshaped, changing dramatically from interphase to mitosis. The dynamic behavior of microtubules is regulated by associated proteins that can stabilize microtubules as required to form the mitotic spindle and other structures (reviewed in [2,3]).

Although microtubules can form spontaneously from high concentrations of tubulin *in vitro*, in cells they are nucleated by specialized microtubule-nucleating proteins, some of which are associated with the centrosome. The centrosome is composed of two barrel-shaped microtubule-based organelles, the centrioles, surrounded by proteins collectively called the pericentriolar material (PCM). Proteins of both the centriole and the PCM can nucleate and anchor microtubules (reviewed in [2,3]).

The role of the centrosome in directing cellular protein traffic depends upon the intrinsic polarity of microtubules, and microtubule-associated motor proteins that move differentially towards one microtubule end or the other. By nucleating microtubules, the centrosome thus determines the tracks along which different cellular components can be transported to different parts of the cell. It can also help to define the speed at which components move along those tracks, and act as a signaling center to modify some components before they are transported to their destinations.

## What’s special about centrioles that distinguishes them from other microtubule-based structures?

**Figure 1:**
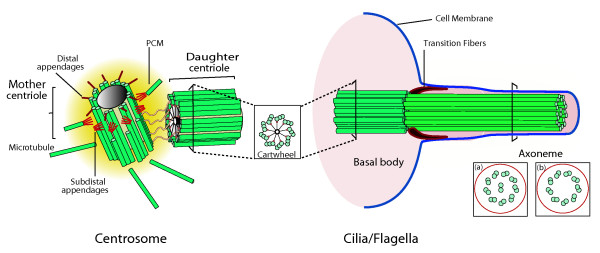
**The structure of centrosomes and cilia/flagella.** The centriole, also called the basal body, is a structural constituent of centrosomes, cilia and flagella. The canonical centriole has nine microtubule triplets and is approximately 0.5 μm long and 0.2 μm in diameter. Each centrosome is composed of a mother (or grandmother) and daughter centriole present in an orthogonal configuration and surrounded by a matrix of proteins called the pericentriolar material (PCM). The older centriole (mother) shows subdistal appendages, where microtubules are docked, and distal appendages, which are important for docking to the cell membrane. In many cells the centriole migrates and tethers to the cell membrane via its appendages and seeds the growth of cilia and flagella. The skeleton of cilia and flagella, called the axoneme, results from a continuation of the basal body structure and might be composed of nine microtubule doublets with dynein arms and a central microtubule pair, as it is for most motile cilia (a); or nine doublets with no dynein arms or central pair, as it is in the case of most immotile cilia (b). The distal part of the basal body is called the transition zone, where the outer tubule stops growing. Adapted with kind permission from Springer Science + Business Media: *Cell Mol Life Sci* Centrioles: active players or passengers during mitosis? *67* (2010). 2173–2194. Debec A, Sullivan W, and Bettencourt Dias M, Figure 1, Copyright © The Author(s) 2010.

They have a very characteristic structure, being composed in most cases of nine triplets of stable microtubules in a small cylindrical arrangement approximately 0.5 μm long and 0.2 μm in diameter. As well as their nine-fold symmetry, the highly conserved structural properties of centrioles include the presence of appendages, a defined size, high stability and ability to recruit PCM components (Figure 1). Centriolar characteristics determine most properties of the centrosome, including dynamicity, polarity and duplication. When tethered to the membrane centrioles are called ‘basal bodies’, structures that provide the template for the formation of the axoneme, the core structure that provides rigidity and mobility to cilia and flagella (Figure 1). Centriole properties also determine the nine-fold symmetry of cilia and flagella, as well as their polarity and localization and orientation at the membrane (reviewed in [2,3]).

The nine-fold symmetry of centrioles is in part provided by the cartwheel, one of the first centriole structures that is assembled upon their biogenesis and that displays that symmetry (Figure 1) [4]. The cartwheel is composed of several components including Sas6 (Spindle assembly abnormal protein 6), which localizes to the cartwheel center, forms oligomers that assemble into a cartwheel-hub-like structure *in vitro*, suggesting how the quaternary structure of that protein defines centriole organelle symmetry [2,3]. This process is likely to be regulated by other molecules, such as Ana2 (Anastral spindle 2 protein)/SAS5 (Spindle assembly abnormal protein 5)/STIL (SCL/TAL1 interrupting locus) (reviewed in [4,5]).

Centriole microtubules are very stable; unlike other microtubules, they are cold and detergent resistant. When labeled tubulin is injected into cells, only daughter centrioles incorporate the label over a period of one cell cycle. Several centriole-specific microtubule-binding proteins, such as Bld10/CEP135 (Centrosomal protein of 135 kDa), SAS4 (Spindle assembly abnormal protein 4)/CPAP, Centrobin and POC1 (Proteome of the centriole protein 1) contribute to both stability and elongation of centriole microtubules. The overexpression of these molecules leads to longer centrioles. Other molecules, such as a depolymerizing kinesin, destabilize them. Centriole stability may also be aided by post-translational modifications of centriolar tubulin, such as glutamylation (reviewed in [5,6]). Centriole size is highly stable and homogeneous after reaching final length, suggesting that a length-maintenance mechanism must exist. Little is known about length regulation, but it has been suggested that a cap may exist at the distal part of centrioles that regulates their length, through the regulation of microtubule nucleation/stabilization. The centriole component CP110 is a strong candidate for this function: it localizes to centriole tips and its depletion leads to the formation of abnormally long centrioles that might fragment originating abnormal mitotic spindles (reviewed in [5,6]).

## What about the pericentriolar material? How do centrioles recruit it and what does it do?

Several centrosome components have been identified recently, through proteomic studies or functional genomic analysis and their localization and function characterized [7-9]. These include CEP192/SPD2 (Spindle-defective protein 2), CEP152/asl (asterless in *Drosophila*), Pericentrin, SAS4/CPAP and CNN (centrosomin)/CDK5RAP2 (CDK5 regulatory subunit-associated protein 2), which bind to centrioles and/or to each other and recruit microtubule nucleators, such as gamma-tubulin. From these studies, a new view, of a highly organized PCM is emerging, where different domains might be involved in separate functions and are regulated differently through the cell cycle [10-14]. The size and organization of the PCM is likely to impinge on centrosome function and is determined by the intrinsic properties of its components (size, shape and protein domains, amongst others), their availability and their regulation by kinases [5,9,15]. How this all works to ensure centrosome function is poorly understood and is an important avenue of research for the future.

## Aren't centrosomes essential for all cells?

**Figure 2:**
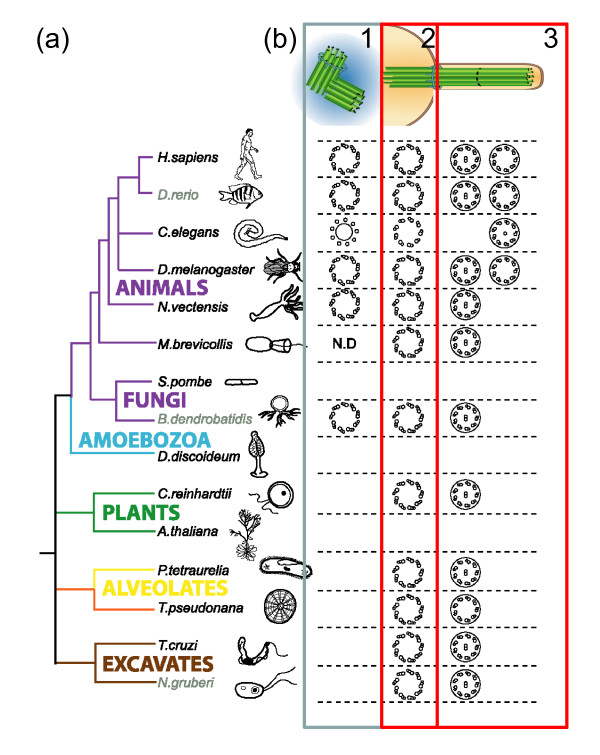
**Presence of centriole/basal body structure correlates with the presence of flagella/cilia. (a)** Simplified taxonomic tree representing major eukaryotic groups in different colors. Unikonts include eukaryotic cells that, for the most part, have a single emergent flagellum divided into opisthokonts (propel themselves with a single posterior flagellum; metazoans, fungi and choanoflagellates) and Amoebozoa. Bikonts include eukaryotic organisms with two emergent flagella. Branch color code: purple, opisthokonts; blue, Amoebozoa; green, plants; yellow, alveolates; orange, stramenopiles; rose, Rhizaria; brown, excavates and discicristates. Adapted with permission [19]. **(b)** We represent the symmetry and number of microtubules present in centrioles/basal bodies that are either nucleating (basal body, 2) or not nucleating axonemes (centriole within centrosome, 1) and in axonemes (3) as well as the presence/absence of a central microtubule pair. Note that the presence of the centriole/basal body structure (2) correlates with the presence of flagella (2 versus 3) but not centrosomes (1 versus 2). Adapted from © Carvalho-Santos et al., 2011. Originally published in *J Cell Biol*.

**Figure 3:**
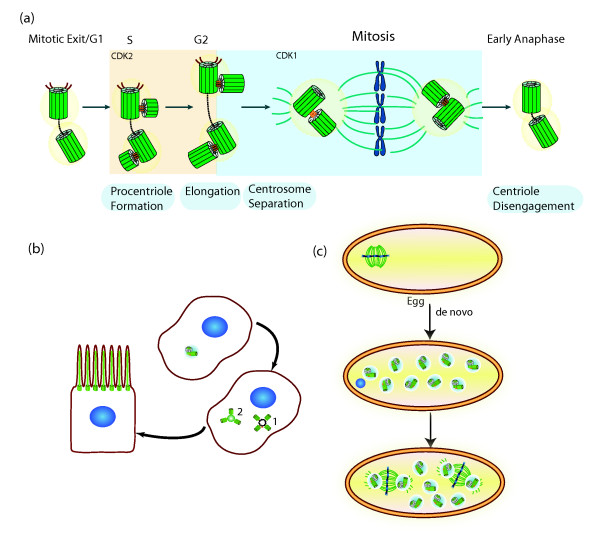
**Regulation of centrosome number. (a)** The canonical centrosome cycle. Procentriole formation begins in S phase orthogonally to its mother. CDK2 activity may be necessary for speeding up procentriole formation and elongation, thus coordinating this event with DNA replication. In G2, the daughter centriole reaches full elongation and maturation with the recruitment of several molecules to the pericentriolar material (PCM). CDK1 activity increases in G2 regulating a variety of molecules and processes needed for entry into mitosis, such as changes in microtubule dynamics. Through the concerted action of molecules such as the kinase Nek2, the two centrosomes separate. The mitotic spindle segregates the chromosomes equally to the two daughter cells. When a cell exits mitosis, the centrioles within the centrosome disengage. That process may allow recruitment or activation of molecules necessary for duplication. Adapted with kind permission from Springer Science + Business Media: *Cell Mol Life Sci* Centrioles: active players or passengers during mitosis? *67* (2010). 2173–2194. Debec A, Sullivan W, and Bettencourt Dias M, figure 4, Copyright © The Author(s) 2010. **(b)** Formation of multiple centrioles during ciliogenesis of epithelial cells. Hundreds of centrioles are formed either around a pre-existing mother centriole (1) or a deuterosome (2). These centrioles migrate and dock to the cell membrane, where they nucleate cilia. **(c)***De novo* centriole formation during oogenesis of parthenogenic insect species. Centrioles disappear during oogenesis in many animal species. Female meiosis is acentriolar. After egg activation multiple centrioles arise *de novo* and join the female pronucleus resulting from meiosis. In the absence of fertilization, those MTOCS set up the first mitotic spindle in the unfertilized egg. The remaining MTOCs disappear. Adapted with permission from John Wiley and Sons: Cunha-Ferreira I, Bento I, and Bettencourt Dias M. Traffic. Copyright Journal compilation © 2009 Blackwell Munksgaard.

No. Centrosomes are not essential in somatic cells in fruit flies, and many animal cells don’t have them (reviewed in [16]). Most eukaryotic cells do have a microtubule cytoskeleton but this can be organized in many different ways by MTOCs, which need not be centrosomes. Several species do not have centrosomes. In others, centrioles and/or centrosomes are absent or inactive in some tissues, while they can exist in very high number in others. In many cells in *Drosophila,* centrosomes are inactive in interphase and only become active in mitosis [17]. Centrosomes are absent in many species of fungi and seed plants, as well as in many classes of protists, and in these species the specific genes encoding the proteins responsible for the nine-fold symmetry of centrioles, appendage formation, microtubule stability and length regulation have been lost [18,19] (Figure 2). Within animals, the flatworm *Planaria*, despite making centrioles that assemble cilia, does not have centrosomes [20]. Moreover, even within mammals there are cases of acentriolar cells: female oocytes lack centrioles (Figure 3c) and the mouse embryo develops with no centrioles until the 64-cell stage [21]. Commonly, in differentiated animal cells the centrosome is no longer the major MTOC and is inactive. That is the case for muscle cells, epithelial cells and neurons. In those cell types, upon differentiation, the centrosome often loses PCM components, which delocalize to other parts of the cell such as the cytoplasmic membrane and the nuclear envelope, which then behave as the MTOC (reviewed in [16,22]). Furthermore, centrosomes are not essential for mitotic spindle assembly, even in cells that normally have them. Moreover, mutant flies that do not assemble centrosomes from centrioles or that do not have centrioles can develop to adulthood [23,24]. In those flies, somatic cell division is fine although some defects are observed in asymmetric cell division and cytokinesis [24]. In summary, centrosomes contribute to mitotic fidelity, cytokinesis and asymmetric cell division, but this is not essential for the development of the flies (reviewed in [16]).

## Are there centrosome-independent mechanisms involved in spindle assembly?

In recent years it has been shown that several cooperative strategies contribute to nucleate and/or stabilize microtubules for the spindle. The chromatin pathway generates microtubules close to the chromosomes, a process that can depend on RanGTP or a molecular complex called CPC (chromosome passenger complex). Moreover, microtubules can be nucleated from pre-existing microtubules, through a molecular complex called augmin. Finally, the nuclear envelope may also contribute to microtubule nucleation (reviewed in [25]). Therefore, it would appear that centrosomes are not always necessary for spindle assembly and cell division.

## Are centrosomes ever important for cell division?

Centrosomes are important for specialized cell divisions. For example, in *Drosophila*, adult males with no centrosomes show highly abnormal meiotic divisions [26]. Moreover, eggs from mothers that are mutant for centriole proteins arrest very early in embryonic development after only a few abnormal mitoses, showing that centrioles are necessary for syncytial mitoses [26,27]. Moreover, asymmetric cell divisions can also be abnormal in the absence of centrosomes (reviewed in [16]). In summary, whereas centrioles may be dispensable for cell division in some tissues of the fly, they are absolutely essential in others, perhaps due to tissue specificity constraints, such as weaker checkpoints, different cell size and/or sharing of common cytoplasm in the context of a syncytium. The same is true in other organisms, such as the *Caenorhabditis elegans* embryo and fission yeast, where the centrosome and its equivalent, the spindle pole body, are essential for bipolar spindle assembly and cytokinesis, respectively (reviewed in [16,26]).

## So if it’s not strictly necessary for spindle formation, what is the significance of the positioning of the centrosome at the poles of the mitotic spindle?

As early as 1887, centrosomes were seen at the poles of the mitotic spindle, which led to their identification as 'the organ for cell division’ by Boveri and Van Beneden. A large debate exists on whether this is just an epiphenomenon (reviewed in [4]). As mentioned above, centrosomes are absent from varied eukaryotic organisms and cell types, suggesting they are not essential for spindle assembly (Figure 2). Pickett-Heaps said that: ‘The centrioles instead appear more likely to be inert passengers ensured of being partitioned equally between daughter cells by being attached to the spindle apparatus’. Friedlander and Warhman proposed that ‘the spindle of Metazoan cells is a basal body distributor that guarantees the accurate segregation of both chromosomes and centrioles (basal bodies)’ (reviewed in [16,28]). Could this be the case? These arguments would suggest that the most important function of the centriole is to form flagella/cilia and not centrosomes; that centrosome-independent mechanisms are involved in spindle assembly and that the centrosome is a modification of centrioles that only localizes centrioles at the poles of the spindle to ensure equal distribution to daughter cells.

The analysis of the distribution of centriole/basal bodies, cilia/flagella and centrosomes through the eukaryotic tree of life shows there is a strict correlation between the presence of centrioles and cilia/flagella; however, the correlation is poor between the presence of centrioles and centrosomes, as the presence of the latter is more limited in the eukaryotic tree of life (Figure 2). For example, some species only form centrioles when they form cilia/flagella, such as the amoeboflagellate *Naeglaeria*, mosses or *Planaria*, in which case they form them *de novo*. This supports the idea that the ancestral and most important function of centrioles/basal bodies is indeed in cilia/flagella assembly, and not centrosome assembly, and suggests that centriole location at the poles is an epiphenomenon (reviewed in [18]). However, it is likely that once at the spindle poles, centrosomes might have acquired new functions in different eukaryotic cells and different cell types within animals, as discussed in the previous question (reviewed in [18,26]).

## What is the evidence for the formation of the centrosome being a strategy for equal distribution of basal bodies to the daughter cells?

The localization of centrioles at or close to the poles of the spindle is often achieved through the interaction of microtubules nucleated by the centrioles and the spindle itself. The two centrioles in the centrosome remain associated through mitosis. The absence of a centrosome in *Planaria* provides food for thought. In planarians, centrosomes are never found at the poles of the spindle [20]. Centrioles are only present in epithelial cells, where they are assembled *de novo* and build motile cilia after anchoring at the cell membrane. Remarkably, *Planaria* lost from its genome several genes that are known to be involved in forming a centrosome; that is, in endowing the centriole with the ability to nucleate microtubules [20]. That is the case of CNN/CDK5RAP2, SPD2/CEP192, centrobin, and NEK2 (NIMA (never in mitosis gene a)-related kinase 2); the first three genes are important for centrosome maturation, which contributes to centrosomes' ability to generate microtubules that capture the spindle (reviewed in [2,9,29]). A consequence of their depletion is that cells inherit abnormal centriole numbers [23,30,31]. Furthermore, it is known that NEK2 is involved in centrosome separation, a process that is necessary for centrosomes to localize to opposite spindle poles at the entry to mitosis [32]. The above arguments suggest that the localization of centrioles at or close to spindle poles via direct microtubule nucleation or through binding to a MTOC is likely to be a strategy to ensure equal inheritance of these structures. However, given that the centrosome plays important roles in cell division in several organisms and tissue types, it is possible that it has been co-opted to actively participate in spindle assembly in certain contexts [18].

## What controls the number of centrioles in a cell?

Different cells have different numbers of centrioles. While, as discussed above, most oocytes have no centrioles, in mammalian epithelial multiciliated cells, such as the ones of the vertebrate respiratory system, 200 to 300 basal bodies are formed in each cell after differentiation. Multiple centrioles form around a mother centriole, differing from the usual pattern of one daughter centriole per mother centriole. Centrioles can also form around less characterized, non-microtubule-based dense structures of heterogeneous size, called deuterosomes, whose composition is unknown (reviewed in [4,16,22]) (Figure 3b). In a dividing cell the number of centrosomes is highly controlled through a canonical duplication cycle in coordination with the chromosome cycle (Figure 3a): one centriole forms per mother centriole in each cell cycle. Four structural steps were defined through electron microscopy in the canonical cycle: separation (called disengagement) of the centrioles, formation of the daughter centrioles close to the mother, elongation of the daughters and separation of the centrosomes in G2 (reviewed in [5,6,9]; Figure 3a).

While a cell in G1 has one centrosome, during the rest of interphase and mitosis it has two, with each centrosome harboring two visible centrioles from S phase. In G2 the two centrosomes separate and their presence as individual entities becomes more obvious. Thus, when the cell enters mitosis it is equipped with two centrosomes, each with two centrioles, which participate in mitotic spindle assembly. The centriole cycle is regulated by the same machinery that regulates the chromosome cycle, such as cyclin-dependent kinases (Figure 3a). How those molecules regulate centriole components is not known (reviewed in [5,6]).

Several remarkable rules regulate centriole number and localization in canonical centrosome biogenesis: it occurs once per cell cycle, only one daughter is formed per mother, and no centrioles are formed away from the mother. Once centrioles have duplicated in S phase, they cannot duplicate again until the next S phase. Disengagement of the centrioles at the exit of mitosis is a prerequisite for duplication in the next cell cycle and much work is now focused on understanding this step. Little is known about the control that ensures that one and only one daughter centriole forms close to each mother. However, overexpression of some regulators, such as PLK4, SAS6 and Ana2/SAS5/STIL, can override that control [5,6].

Centrioles can also appear without a pre-existing centriole (*de novo* formation). *De novo* biogenesis is known to occur in insect species with parthenogenic development, as well as in human cells upon laser ablation of their centrosomes or overexpressing some centriole regulators (Figure 3c). The localization and number of these centrioles is not determined and can change significantly. Clearly the *de novo* pathway is regulated by the same molecules as the canonical pathway, however it has to be very well controlled to avoid multiple centriole formation in normal cells [5,6,9].

## What determines the different roles of centrioles?

Centriole age, and in consequence centrosome age, might be physiologically and developmentally very important. A consequence of the centriole cycle is that each centrosome in a mitotic cell has a different age: one has a mother and a daughter, the other a grandmother and a daughter centriole. These differences provide variation in the competence for PCM acquisition, microtubule nucleation, anchoring and cilia formation. After cytokinesis the cell inheriting the grandmother, appendage-harboring centriole, grows the primary cilia first and is thus able to respond to signaling cues, which may generate asymmetry amongst those cells [33,34].

The age of centrioles also biases their ability to be retained differentially in *Drosophila* male germ line stem cells and neuroblasts, and rodent neural progenitors, and could be implicated in proliferation and fitness of stem cell niches and/or progenitor cells, with consequences in development and morphogenesis (reviewed in [35,36]). This topic deserves more attention, as a recent study has shown that randomization of centrosome inheritance does not affect asymmetric cell division [37]. How the age of a centriole affects its ability to be retained in one cell versus another is a very interesting question. One possibility is that centriole microtubule-nucleating and microtubule-anchoring capacity defines the population of astral microtubules associated with that centrosome and this may provide different connections with the cell cortex. Indeed, the age of the centriole determines the presence of particular proteins at each centriole, which then determines their specific microtubule nucleating capacity and centrosome inheritance [37].

## Centriole and centrosome functions are clearly critical to many processes - what happens when they don’t work properly?

A variety of human diseases have been linked to centrioles and centrosomes, such as diseases of brain development, cancer and ciliopathies. The wild-type product of the mutated gene often localizes to centrosomes and/or has a centrosome function (for example, CPAP, CDK5RAP2, CEP152, STIL, amongst others; reviewed in [38,39]). The most common phenotypes in brain disorders associated with those proteins are generalized disorders of growth where the brain is disproportionately affected; and the primary microcephalies where the brain alone is affected and significantly reduced in size. One current hypothesis for the latter is that centrosomes help in spindle positioning in neural progenitors, contributing to a balance between expansion of progenitors and generation of neurons. It is equally possible that some of the divisions with abnormal centrosomes might lead to aneuploidy and cell death. Animal models of the human mutations associated with those diseases should play an important role in the understanding of their genesis (reviewed in [38,39]).

With respect to cancer, Boveri, Hansemann and Galeotti, more than a century ago, proposed that abnormalities in centriole duplication could be at the origin of the genome instability observed in cancer cells [39,40]. There is an extensive debate on whether centrosome defects commonly observed in cancer, such as supernumerary centrosomes and/or centrosomes with altered structure, are a by-product of mitotic abnormalities or if they actively contribute to tumorigenesis. Centrosome abnormalities can occur early in pre-malignant lesions and are extensively correlated with aneuploidy, supporting a direct role for extra centrosomes in tumorigenesis [40]. Moreover, the presence of abnormally high numbers of centrosomes per cell can generate tumors in flies [41]. How can abnormally high numbers of centrosomes generate cancer? Cancer cells adapt to dividing in the presence of supernumerary centrosomes by clustering them at the poles of a bipolar spindle; however, in the process of organizing a bipolar spindle those cells may generate abnormal chromatid attachments that lead to aneuploidy (reviewed in [39,42]). Extra centrosomes can also interfere with asymmetric cell divisions, which may lead to hyperproliferation [41] (reviewed in [39]). Supernumerary centrioles may also generate supernumerary cilia, which lead to abnormal ciliary signaling (for example, hedgehog), at least in tissue culture cells [33].

What about ciliopathies? Cilia can be motile or immotile, such as those of specialized cells like photoreceptors, and of primary cilia, which are sensing structures that exist in most human cells. Motile cilia assembly defects were first associated with bronchitis, sinusitis, and sperm immotility. Changes in body symmetry have shown that ciliary motility is essential to create directional flow in the early embryo, initiating the normal left-right developmental program. In recent years, a variety of syndromes were included in the ‘ciliopathies’ list, where mutations in genes whose product localizes at the primary cilia and/or centrosome lead to abnormal ciliary structure and/or function. This is the case of several rare disorders, such as polycystic kidney disease, nephronophthisis, retinitis pigmentosa, and Bardet-Biedl, Joubert and Meckel syndromes. The study of those proteins is contributing to a better understanding of the function of immotile cilia. In particular, in several of those diseases the microtubule-based structure of the cilia is not altered, while its sensory function might be [39,43].
